# The Effect of Irrigation with Citric Acid on Biodentine Tricalcium Silicate-Based Cement: SEM-EDS In Vitro Study

**DOI:** 10.3390/ma15103467

**Published:** 2022-05-11

**Authors:** Katarzyna Dąbrowska, Aleksandra Palatyńska-Ulatowska, Leszek Klimek

**Affiliations:** 1 Department of Dental Techniques, Chair of Restorative Dentistry, Medical University of Lodz, 251 Pomorska Street, 92-217 Lodz, Poland; katarzyna.bula@stud.umed.lodz.pl; 2 Department of Endodontics, Chair of Conservative Dentistry and Endodontics, Medical University of Lodz, 251 Pomorska Street, 92-217 Lodz, Poland; 3 Institute of Materials Science and Technology, Technical University of Lodz, 1/15 Stefanowskiego Street, 90-924 Lodz, Poland; leszek.klimek@p.lodz.pl

**Keywords:** citric acid, energy dispersive spectroscopy, irrigation, perforation, scanning electron microscope, ultrasonic activation

## Abstract

There are various factors that may interfere with the activity of biomaterials during endodontic therapy. One of them is the canal system irrigation procedure with different rinsing solutions performed after the placement of bioactive cements. The authors investigate the influence of citric acid, a chelating agent, on the surface and the chemical composition of Biodentine tricalcium silicate-based cement using a multimethod approach. Twenty samples were divided into two groups based on the material setting time. They were subjected to citric acid irrigation with or without ultrasonic activation for 5 and 20 min. The chemical analysis was made with energy dispersive spectroscopy (EDS). The visual assessment of Biodentine surface was carried out in scanning electron microscope (SEM). The volume of material loss during the procedure was measured with Keyence optic microscope and dedicated digital software. Statistical analysis was performed. The results of the study show that the irrigation with citric acid influenced the surface appearance of the material and changed its chemical composition in both investigated groups. The ultrasonic activation (US) of the liquid has also aggravated its impact. Further research is needed to assess if that fact may change the sealing properties of the material influencing the long-term clinical outcome.

## 1. Introduction

Biomaterials in endodontic reparative treatment are often the last resort for tooth retainment. They are especially designed to effectively seal the defect and biologically fit in into wounded place contacting periodontium, e.g., in furcal perforations or intraosseous environment [1]. Mineral Trioxide Aggregate (MTA), a Portland cement-based material, has been successfully used for this purpose for over 25 years [2]. Nowadays, new types of so-called bioceramic materials are emerging on the market. Their chemical composition, hence, the therapeutic properties, are a key to high overall treatment success rate. The features such as biocompatibility, antimicrobial activity, hydraulic properties, push-out-bond strength and release of calcium hydroxide are of equal importance. Therefore, clinicians’ awareness of these materials’ nature, ability to properly choose, use and manage them in different clinical situations need to be constantly recalled and renewed.

Biodentine (Septodont, Saint-Maur-des-Fosées, France) is one of commonly used reparative materials in endodontic therapy. It is known as “dentin replacement” self-setting hydraulic calcium-silicate-based cement [3,4,5,6,7]. It has numerous clinical applications such as pulp capping [8,9,10], pulpotomy procedures [11,12,13], treatment of an immature or open apex teeth [2,13], in root-end retrograde surgical fillings [2,14] and perforation repair [2,15,16].

The tricalcium and dicalcium silicate phase is mainly responsible for the bioactivity of Biodentine [17]. The other components are zirconium oxide as a radiopacifier, calcium carbonate and calcium oxide as fillers, calcium chloride as setting reaction accelerator and hydrosoluble polymer as water requirement reductor [18]. Its chemical composition supports biocompatibility, faster setting time when compared to MTA [2,19] and good sealing properties [20].

The cleaning and shaping of the endodontic space includes a thorough chemical preparation protocol. The most often used rinsing solutions are 5.25% sodium hypochlorite, 2% chlorhexidin, 15% EDTA or, interchangeably, 40% citric acid (CA). Also, the canal preparation time with intense irrigation in relation to the placement of the bioactive material may vary and depends on the performed procedure. However, the decision about the order and the course of each treatment step may become a challenge. In root perforation, which is a communication between canal system and the external tooth surface [21], either as a pathological process or as a procedural accident, it is crucial to close it as quickly as possible after its occurrence. The immediate diagnosis and proper, instantaneous treatment largely influence the prognosis [22,23]. In some clinical situations, mainly iatrogenic, Biodentine is placed before the end of chemo-mechanical preparation. In most cases, a thorough canal debridement is achieved by mechanical and chemical means prior to cement placement. However, large, bleeding perforations that impede canal shaping or some reendo cases with fresh chamber floor perforations require early closures in order to avoid their contamination and pushing the debris of removed filling from infected canals through the perforation aperture. As a consequence, the cement used for this procedure has to withstand the forces and factors connected with the standard endodontic therapy. The material resistance is dependent on its setting stage and on technical aspects of mechanical shaping, irrigation and, finally, chemical removal of the smear layer created throughout the procedure. Thus, the continuation of endodontic treatment may negatively impact the clinical performance of freshly placed perforation closure cement [24].

The study concerns citric acid rinsing solution used during the final irrigation protocol. It is a chelating agent that reacts with metals and forms a non-ionic soluble chelate. This feature enables citric acid to remove the inorganic part of the smear layer [25]. It helps to expose a large number of dentinal tubules and increase the contact area for better adaptation between the sealer and root canal dentin [26,27]. The chelating nature of citric acid makes it a possible threat to any kind of biocement placed within the root canal system.

The standard rinsing procedure is performed with a syringe, nevertheless various activation techniques have been proposed in order to improve the activity of the irrigation liquid. One of the most researched and known is the Passive Ultrasonic Irrigation (PUI). The vibration of the ultrasonic tip produces an acoustic stream able to dislocate debris from canal walls and physically rupture the bacterial aggregations [28]. The whole process is improved by microcavitation and the irrigant solution temperature rise [29,30].

The aim of the study is to analyze the influence of citric acid used during endodontic procedures, with or without ultrasonic activation, on the chemical composition and surface appearance of Biodentine cement. In the context of a lack of available literature on this topic, the research is the first of its kind.

## 2. Materials and Methods

**Figure 1:**
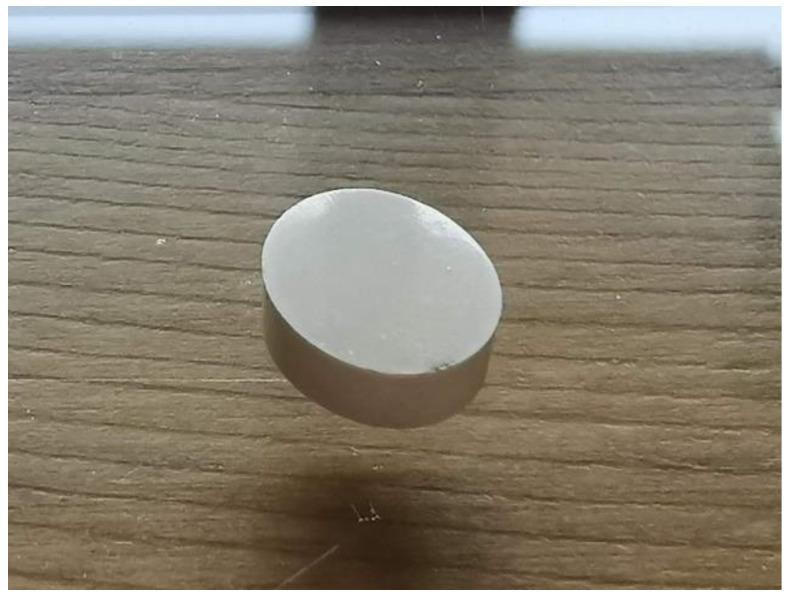
Biodentine sample (8 × 3 mm) removed from the PVC form.

Twenty standardized Biodentine disc-shaped samples were prepared according to the manufacturer’s instructions. Exact guidelines were respected regarding powder and liquid proportion, time and mixing method (Dental Mixer SYG200, Septodont, Saint-Maur-des-Fosées, France). Prepared cement was placed into a polyvinyl tube (polyvinyl chloride—PVC; Cellfast, Stalowa Wola, Poland) and compacted with a fitted plugger for 5 s on a smooth glass plate. The size of the cylindrical samples was 8 mm in diameter and 3 mm in height (Figure 1).

Biodentine was then left to set for a determined amount of time measured form the end of mixing process: 45 min [31,32] and 24 h. Samples were divided into two groups: A- after 45-min setting time and B—after 24-h setting time. The specimens were delicately removed from the PVC forms with customized plugger and their investigated surface was dry-polished with rotating sandpapers (following grits of 600, 800, 1000 and 1200). After being categorized randomly inside of the two setting-time groups they were subjected to irrigation with 40% citric acid (Cerkamed, Stalowa Wola, Poland) with or without ultrasonic activation (US, ultrasounds): samples were immersed in 10 mL of CA for an exact time of 5 and 20 min. They were either left without any further activity or the liquid was ultrasonically activated throughout the whole irrigation time in a device Sonic-0.5 (50 Hz frequency, Polsonic, Warsaw, Poland). In order to avoid any precipitates on the evaluated surfaces, each sample, after being removed from the irrigation container, was rinsed with demineralized water, the excess of which was blown away with compressed air.

**Figure 2:**
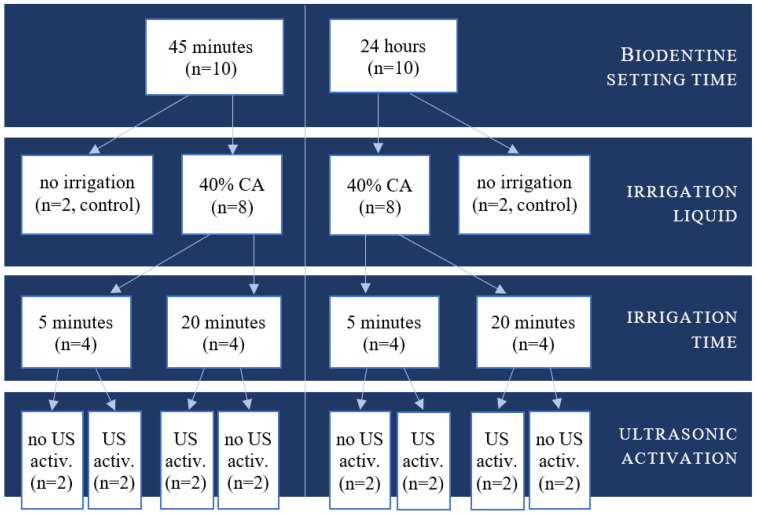
Schematic presentation of Biodentine samples’ distribution.

The control group included 4 samples after 45-min and 24-h setting time and it was not subjected to any irrigation protocol. The distribution and number of Biodentine control and examined samples is shown on Figure 2.

Scanning electron microscope investigation (SEM, S–3000N, HITACHI, Tokyo, Japan) and elemental analysis of 20 samples were performed from the area of 2 × 3 mm with magnification of 1.0 k, 15 kV accelerating voltage and 15 mm working distance. The Biodentine chemical composition analysis of the surface was conducted in SEM using the X-ray microanalysis with the energy dispersive spectroscopy method (EDS) using the Vantage software (Thermo Fisher Scientific, Waltham, MA, USA).

**Figure 3:**
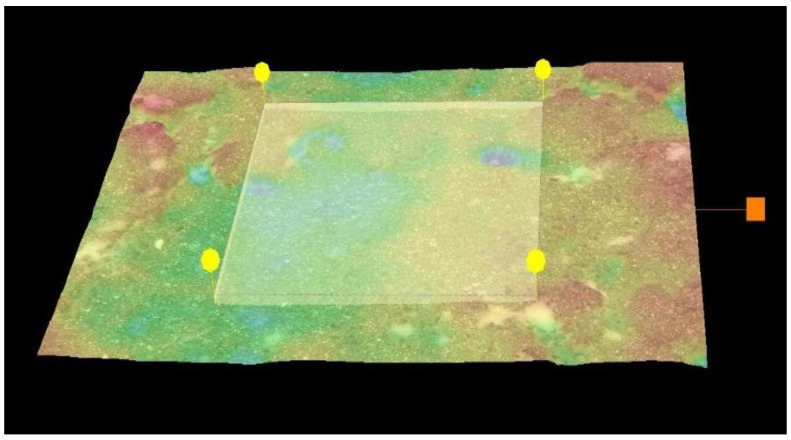
Volume measurement in optic microscope VHX-950F (Keyence).

The volume of material loss was evaluated under 0.5 k magnification in optic microscope VHX-950F with the digital software (Keyence, Mechelen, Belgium). The measures were taken in 5 random locations on each specimen. The exemplary measurement area is shown on Figure 3.

The statistical analysis was performed to compare the influence of three factors: Biodentine setting time, irrigation time and ultrasonic activation on material volume loss. Results were presented as means with standard deviations. Distribution of continuous variables was evaluated using Shapiro-Wilk test. Comparison between dependent variables was made using t-Student test or Wilcoxon signed rank test. Additionally, test power was calculated for each comparison. A *p* value below 0.05 was deemed significant. All analyses were made using Statistica 13 software (StatSoft, Cracow, Poland).

## 3. Results

### 3.1. Surface Appearance

**Figure 4:**
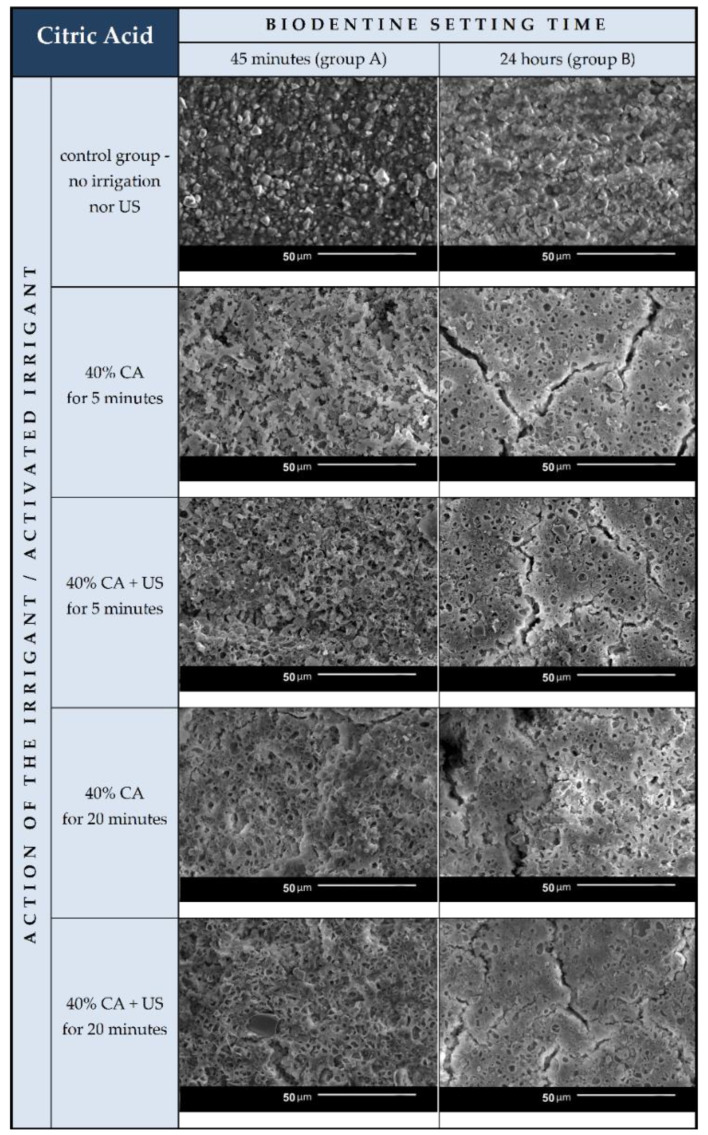
SEM images of the Biodentine control samples without any rinsing procedures and the studied samples after 45-min (group A) and 24-h (group B) setting time subjected to different irrigation protocols. The table also shows the distribution of all groups and evaluated specimens in the study based on the setting time of the material (45 min and 24 h), time of irrigation protocol (5 and 20 min) and the use of ultrasounds (+US).

SEM images of the A and B sample groups were compared to the images of the control group. The comparisons showed that citric acid alone and enhanced with ultrasonic activation visibly affected the Biodentine surface (Figure 4).

In comparison to regular surfaces of control samples, Biodentine surface after CA irrigation in both groups A and B became more irregular. All of the samples after higher time of irrigating liquid action were more affected. Numerous round-shaped defects and hollow pits in most of the specimens resemble the etched dentin with open tubules. They are concave, contrary to the convex unevenness of the control samples.

The ultrasonic activation had more impact on Biodentine in group A—their surface after US activated irrigation was more affected and irregular than after non-activated irrigation. In contrast, ultrasonic activation did not seem to influence the cement in group B. Samples in group A are visually more affected than samples in group B. The quantity and density of sharply edged pits, gaps, and grooves is visibly higher.

### 3.2. Chemical Surface Composition

On the basis of the EDS obtained data, spectrograms were generated (Table 1) and the percentage composition of the evaluated material was registered (Table 2). The results were compared to the control group and between each other.

There are differences in chemical composition of Biodentine samples before and after irrigation with CA with or without ultrasounds. Alterations are also visible when comparing samples of groups A and B.

The most pronounced atom percentage loss concerns calcium. The irrigation with citric acid provokes Ca drop—the longer the irrigation time, the higher the drop. Also, ultrasonic activation aggravates the effect in both irrigation times.

The most pronounced atom percentage rise concerns silicon. Analogically to Ca, the irrigation with citric acid provokes Si increase: the longer the irrigation time, the higher the rise. The ultrasonic activation aggravates the effect in both irrigation times.

Samples from group B are more affected than those from group A. In group A, the calcium percentage drops from 28.87% in control group to 9.1% in Biodentine after CA irrigation with ultrasonic activation, while in group B these parameters are equal to 27.74% and 6.5% respectively. In group A, the silicon level rises from 5.96% in control group to 17.2% in CA irrigated and US activated cement, while in group B it rises from 9.09% to 23.1% respectively.

### 3.3. Volume Loss

**Figure 5:**
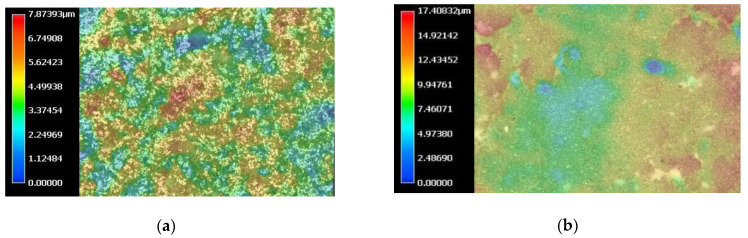

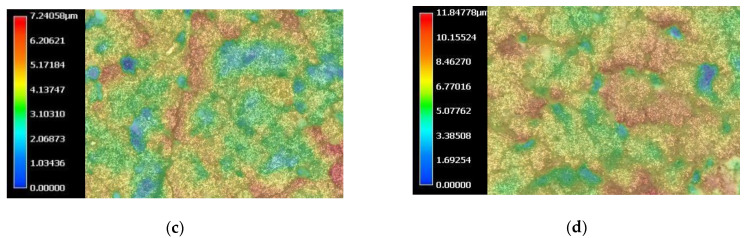
Color 3D map of Biodentine surface (**a**) of the 45-min setting sample after 5 min irrigation with CA, (**b**) of the 45-min setting sample after 20 min irrigation with CA ultrasonically activated, (**c**) of the 24-h setting sample after 5 min irrigation with CA and (**d**) of the 24-h setting sample after 20 min irrigation with CA ultrasonically activated.

Figure 5 presents color 3D maps of borderline samples’ surface.

The mean values of volume loss measured on the area of approximately 96,230 µm^2^ of each sample are shown in Table 3.

The volume loss gets higher and higher within group A with the intensity of irrigation. After 5 min of CA irrigation, it is equal to 5.2 µm^3^ per each µm^2^ of the surface area. After the same irrigation with ultrasonic activation this amount is slightly higher: 7.5 µm^3^. For samples subjected to 20 min irrigation with citric acid, the loss is 26.8 µm^3^ per µm^2^ and the ultrasonic activation raises this value to 39.1 µm^3^.

Within group B, the volume loss is not equally regular, but it is generally higher than in group A. After 5 min of CA irrigation, it is equal to 21.9 µm^3^ per µm^2^ of the surface area. After the same irrigation with ultrasonic activation this amount is slightly higher: 24.4 µm^3^. For samples subjected to 20 min irrigation with citric acid, the loss is 20.1 µm^3^ per µm^2^ and the ultrasonic activation decreases this value to 14.1 µm^3^.

**Figure 6:**
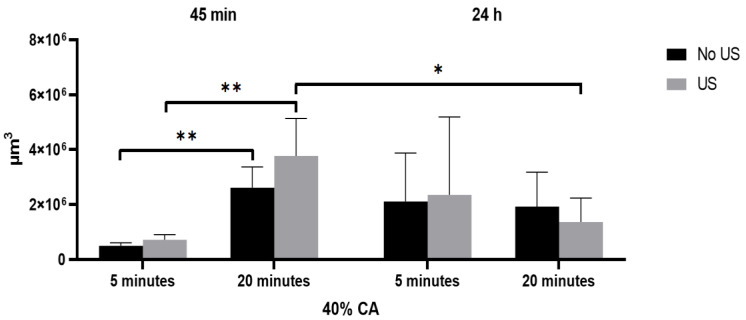
Statistical analysis: Biodentine volume loss depending on material setting time (45 min and 24 h), CA irrigation time (5 and 20 min) with and without its ultrasonic activation. Asterisks above graph indicate level of statistical significance * *p* < 0.05, ** *p* < 0.01.

The results of statistical analysis are presented in Figure 6 and in Table 4 showing the dependance of material volume loss on three factors: Biodentine setting time (a), irrigation time (b), and ultrasonic activation (c).

Statistical analysis indicates that a 20 min irrigation time of a sample from gr B (45 min setting time) causes an increase in volume loss both with and without ultrasonic activation of citric acid. US activation for a 20-min irrigation time resulted in a significant decrease in volume for Biodentine from gr C (24-h material setting time) (from 3,766,818 ± 1,368,663 µm^3^ to 1,362,135 ± 872,413 µm^3^, *p* = 0.0129). US activation did not have a statistically significant effect on material volume loss in any of the tested groups.

## 4. Discussion

Biocompatibility and the sealing properties of the so-called biocements are the most important features determining the quality and prognosis of the treatment. The material management, however, seems to play an equally significant role. For a clinician, the knowledge of the indications, proper management, and application modes of biomaterials are essential for a long-term success, especially in challenging and compromised cases of chamber or root perforations. The research is therefore highly, clinically relevant.

The setting times used for samples’ segregation were investigated by the authors in their last publication [18]. It has been proven that the 12-min setting time of Biodentine recommended by the manufacturer [33] is too short for the cement to withstand the immediate irrigation protocol, especially using ultrasonic activation. While it may be sufficient for treatment of carious defects in conservative dentistry [34,35], the degradation or destruction of cement surface may negatively affect the endodontic therapy outcome. In the current research, the authors decided not to take 12-min setting time into account.

Group A consisted of samples after the 45-min Biodentine setting time given by Grech et al. [32]. After being subjected to CA irrigation, their surface was visually significantly affected, as if the material were not entirely set. In accordance with general cement behavior and standard setting mechanism, this may be true. According to the American Society for Testing and Materials (ASTM), cement setting is a gradual and continuous process divided into stages: the time of initial setting and the time of final setting. The former ends when the paste starts losing its plasticity. The latter occurs when the concrete becomes rigid and it fractures rather than flows as increasing stress is applied [36]. The physical behavior of Biodentine, which derives from Portland cement, seemingly does not differ much from the above description.

In group B, after 24 h setting, the cement matrix became solid, but nonetheless, citric acid was able to dissolve part of it and “pluck” the crystalline forms from their matrix, leaving empty holes, visible on the SEM images. The differences between samples with and without ultrasonic activation in this group did not seem as marked as in group A. It appears as if CA alone was equally destructive for the Biodentine surface as with ultrasounds. A vague conclusion could be drawn that US activation has no impact on samples set for 24 h.

SEM images show numerous cracks in the Biodentine surface, especially in group B. These are most probably the result of the testing procedure. The vacuum in a SEM chamber obtained after the insertion of tested samples for examination might affect the set cement in this particular way. The above phenomenon does not affect the volume microscopy measurements due to controlled choice of tested spots on Biodentine surface. However, the results of chemical composition analysis with EDS method need to be interpreted in a specific way. The EDS technique aligns the results up to 100% in each measurement. This means that the percentage rise of some elements does not necessarily mean its actual increase in number. If some element is removed or decreased in volume it will automatically cause the increase of other elements. In the researched material the loss of calcium is evident due to chemical nature of the irrigant. Therefore, the rise of silicon, previously forming tricalcium silicates in set material, might be simply the outcome of removing a significant amount of Ca from the system. The software needs to equalize the overall percentage to 100% and thus increase the calculated level of Si. Moreover, the calcium is known to be bonded by citric acid. The process of chelation involves the incorporation of a mineral ion or cation into a complex ring structure by an organic molecule, the chelating agent [37]. In this particular case, the calcium cation from calcium silicate is bonded by the citric acid and forms a hydrosoluble complex or chelate.

The surfaces of the samples after 24 h setting time are visually less developed in SEM investigation than the samples after 45 min setting time. Nevertheless, the chemical analysis and volume loss measurements indicate that the overall surface area tackled by the CA irrigation is smaller in Biodentine samples with shorter setting time.

The paper described an in vitro study, which is the main limitation of this research. Nevertheless, a thorough analysis of the push-out bond strength of rinsed Biodentine and its sealing ability constitutes the future direction that we will follow.

## 5. Conclusions

The irrigation with CA, both with and without ultrasonic activation, visibly influenced the surface appearance of Biodentine. It also changed its chemical composition. The ultrasonic activation of the liquid aggravates its impact, especially on samples after 45-min setting time. Immediate or too early CA irrigation is therefore not recommended. Further investigation of the impact of citric acid on the surface of Biodentine is needed to indicate if these alterations have the potential to clinically change the material properties and affect the final treatment outcome.

## Figures and Tables

**Table 1:** Energy dispersive spectrograms of Biodentine control and boundary investigated samples.

Irrigation Protocol	Biodentine Setting Time	
	45 min (Group A)	24 h (Group B)
**Control group**	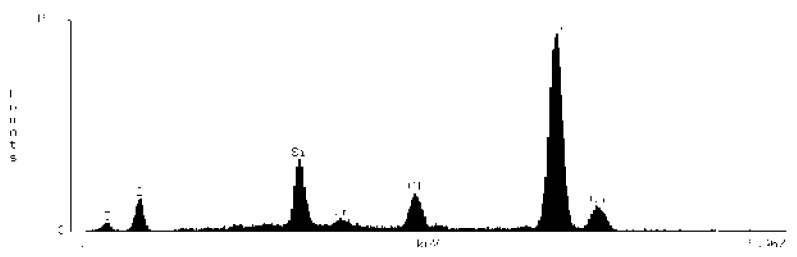	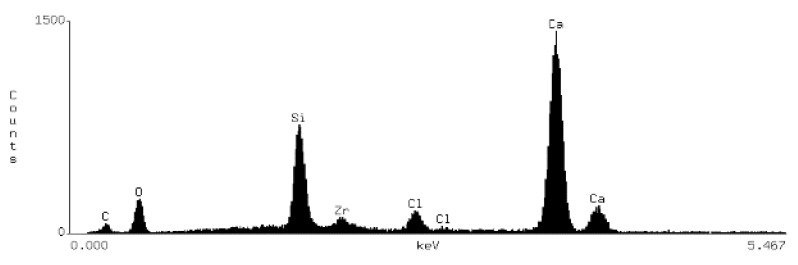
**40% CA;** **5 min**	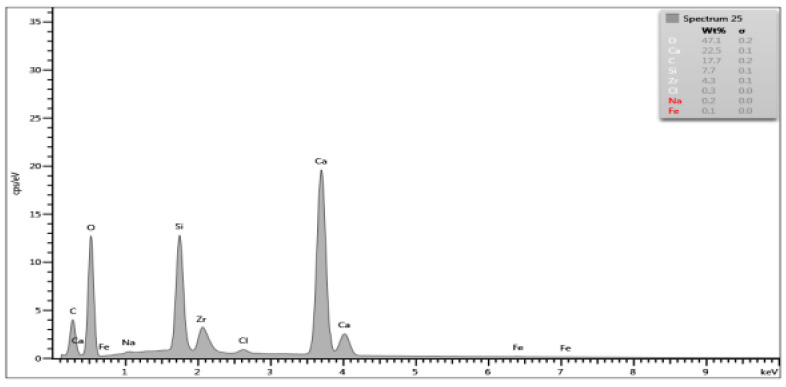	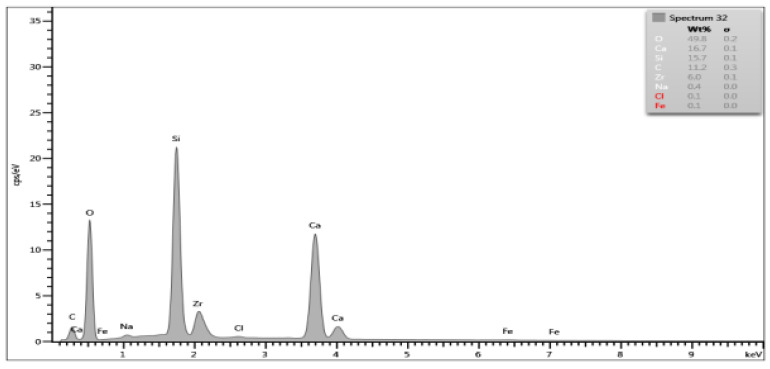
**40% CA + US;** **20 min**	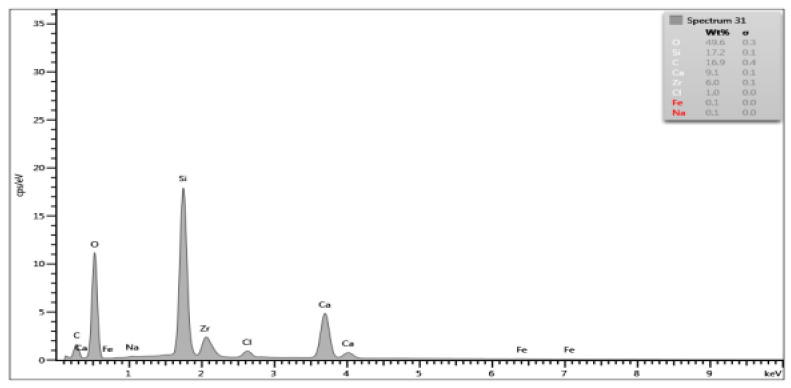	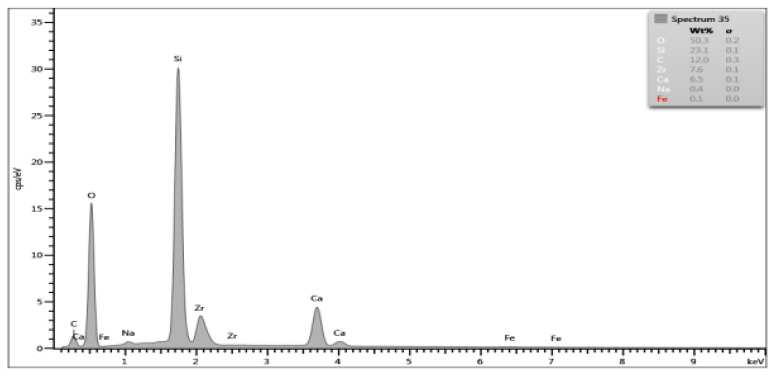

**Table 2:** Chemical composition (atom percentage) of Biodentine samples’ surface after 45-min (group A) and 24-h (group B) setting time.

Elem.	Untreated (Control Group)	40% CA; 5 min	40% CA + US; 5 min	40% CA; 20 min	40% CA + US; 20 min
Group A—atom percentage [%]					
**O**	49.61	47.1	48.7	49.4	49.6
**Ca**	28.87	22.5	14.9	18.9	9.1
**C**	11.35	17.7	12.7	13.2	16.9
**Si**	5.96	7.7	16.7	13.2	17.2
**Cl**	3.64	0.3	0.2	0.7	1.0
**Zr**	0.56	4.3	6.7	4.5	6.0
**Na**	0	0.2	0	0	0.1
**Fe**	0	0.1	0.1	0.1	0.1
**Group B—atom percentage [%]**					
**O**	49.93	49.8	49.6	51.5	50.3
**Ca**	27.74	16.7	18.2	13.2	6.5
**C**	10.41	11.2	9.0	10.5	12.0
**Si**	9.09	15.7	16.8	18.0	23.1
**Cl**	2.09	0.1	0.2	0.3	0
**Zr**	0.74	6.0	5.7	6.1	7.6
**Na**	0	0	0	0.3	0.4
**Fe**	0	0.1	0.1	0	0.1

**Table 3:** The mean values of volume loss (µm^3^) in Biodentine samples after CA irrigation with or without US activation.

Irrigation Protocol	45 Minutes (Group A)	24 Hours (Group B)
40% CA, 5 min	494,610 µm^3^	2,107,092 µm^3^
40% CA + US, 5 min	716,193 µm^3^	2,352,206 µm^3^
40% CA, 20 min	2,608,207 µm^3^	1,920,331 µm^3^
40% CA + US, 20 min	3,766,818 µm^3^	1,362,135 µm^3^

**Table 4:** Statistical analysis—material volume loss depending on (**a**) Biodentine setting time, (**b**) CA irrigation time, (**c**) ultrasonic activation of CA.

*(a) Biodentine Setting Time.*				
Protocol	45 Minutes (Group A)	24 Hours (Group B)	*p*-Value	Test Power
CA, 5 min.	494,610 ± 106,563 µm^3^	2,107,092 ± 1,768,659 µm^3^	0.1080	0.3488
CA + US, 5 min.	716,193 ± 184,030 µm^3^	2,352,206 ± 2,838,760 µm^3^	0.1380	0.1716
CA, 20 min.	2,608,207 ± 761,599 µm^3^	1,920,331 ± 1,257,015 µm^3^	0.4272	0.1370
CA + US, 20 min.	3,766,818 ± 1,368,663 µm^3^	1,362,135 ± 872,413 µm^3^	*0.0129*	0.7390
*(b) irrigation time*				
**Protocol**	**5 min**	**20 min**	***p*-value**	**Test power**
CA, gr B	494,610 ± 106,563 µm^3^	2,608,207 ± 761,599 µm^3^	*0.0048*	0.9941
CA, gr C	2,107,092 ± 1,768,659 µm^3^	1,920,331 ± 1,257,015 µm^3^	0.8831	0.0529
CA + US, gr B	716,193 ± 184,030 µm^3^	3,766,818 ± 1,368,663 µm^3^	*0.0089*	0.9552
CA + US, gr C	2,352,206 ± 2,838,760 µm^3^	1,362,135 ± 872,413 µm^3^	0.4909	0.0923
*(c) ultrasonic activation*				
**Protocol**	**No activation**	**Ultrasonic activation**	***p*-value**	**Test power**
CA, 5 min, gr B	494,610 ± 106,563 µm^3^	716,193 ± 184,030 µm^3^	0.1542	0.4593
CA, 5 min, gr C	2,107,092 ± 1,768,659 µm^3^	2,352,206 ± 2,838,760 µm^3^	0.8927	0.0521
CA, 20 min, gr B	2,608,207 ± 761,599 µm^3^	3,766,818 ± 1,368,663 µm^3^	0.2811	0.2662
CA, 20 min, gr C	1,920,331 ± 1,257,015 µm^3^	1,362,135 ± 872,413 µm^3^	0.4488	0.1029
